# Supplementary material for: Determinants of Clinical Remission in Dupilumab-Treated Severe Eosinophilic Asthma: A Real-World Retrospective Study
Source: Biomedicines. 2025 Sep 30;13(10):2404. doi: 10.3390/biomedicines13102404 (PMC12562138; doi:10.3390/biomedicines13102404)
Supplement: Supplementary file 1 [file biomedicines-13-02404-s001.zip › biomedicines-3809194-supplementary.pdf]

Determinants of clinical remission in dupilumab-treated severe eosinophilic asthma: a real-world retrospective study.

Matteo Bonato[1], MD, PhD; Elisabetta Favero[2], MD; Francesca Savoia[1], MD; Matteo Drigo[1], MD; Simone Rizzato[1], MD; Enrico Orzes[1] PhD; Gianenrico Senna[3] MD, PhD; Micaela Romagnoli[1] MD, PhD

Affiliations:

1) Pulmonology Unit, Ca' Foncello Hospital, Azienda Unità Locale Socio-Sanitaria 2 Marca Trevigiana (AULSS2), Treviso, Italy

2) Internal Medicine 1, Ca' Foncello Hospital, Azienda Unità Locale Socio-Sanitaria 2 Marca Trevigiana (AULSS2), Treviso, Italy

3) Department of Internal Medicine, University of Verona

ONLINE DATA SUPPLEMENT

Population

All patients treated complied with national indications for dupilumab prescription: (a) blood eosinophils ≥150 cell/µl or (b) FeNO > 25 ppm AND (c) reported at least 2 acute exacerbation treated with ≥ 3/days of systemic corticosteroids despite maximal inhalatory therapy (GINA STEP 4-5) in the previous 12 months or (d) reported in the previous 12 months at least 6 months continuative of maintenance oral corticosteroids. Patients with additional type 2 comorbidities (atopic dermatitis and/or nasal polyposis) as well as those dependent on maintenance therapy with oral corticosteroids (OCS), were administered 300 mg of dupilumab every two weeks with a loading dose of 600 mg, while others received 200 mg with a loading dose of 400 mg.

Patient evaluation and variables assessed

Acute exacerbations (AE) were recorded at baseline and at follow up at 6 and 12 months. Acute exacerbations were defined as an acute worsening of asthma symptoms which required a course of oral corticosteroids ≥ 3 days of duration, severe acute exacerbations were defined as an acute worsening of asthma symptoms which required emergency room access or hospitalization. The number of total acute exacerbations were expressed as the rate for month (episodes/month) during the previous year.

Blood eosinophils counts, total serum IgE and fraction of exhaled nitric oxide (FeNO) dosage were considered only if the tests have been taken within 1 month before the visit. Blood eosinophils were expressed as the number of eosinophils for µl of blood (cell/µl; normal values 0-250 cell/µl; Hartl S, Eur Resp J, 2020), total serum IgE as kilo-units for liter (kU/L; normal values 0-114 kU/L; Movérare R, J Med Sci, 2023) and FeNO as part per billion (ppb; 1 ppb = 1 nmol/mol normal values < 25 ppb; Olin AC, Chest, 2007). Of note FeNO was performed only in 22 out of 52 patients.

Pulmonary function tests were performed according to the ERS/ATS guidelines, forced vital capacity (FVC), forced expiratory flow in the 1st second (FEV1), FEV1/FVC ratio and forced expiratory flow over the middle one-half of the FVC (FEF25-75) were considered as variables of the study.

In the clinical history, smoking habit, asthma symptoms onset, occupational exposure and comorbidities were considered as variables. Former smokers were defined as all patients who smoke more than 100 cigarettes in their life and quit smoking more than 6 months before the first evaluation, active smokers were defined as all patients who smoke at the first evaluation or quit smoking within 6 months before the first visit. Never smokers were defined as smoking less than 100 cigarettes in their life. In consideration of the poor agreement regarding the definition of early and late onset asthma, two age cut-offs (age of 18 and 40) were considered. Considered comorbidities included: allergic rhinitis (AR), atopic dermatitis (AD), chronic rhinosinusitis with nasal polyps (CRSwNP), gastro-esophageal reflux (GERD), obstructive sleep apnoea syndrome (OSAS), obesity, bronchiectasies, asthma-chronic obstructive pulmonary disease overlap syndrome (ACOS), hypertension, emphysema, major depression, bronchopulmonary allergic aspergillosis (ABPA), epithelial malignancies, blood malignancies and eosinophilic granulomatosis with polyangiitis (EGPA).

Reported daily anti-asthmatic prescriptions included maintenance high dose inhaled corticosteroids (ICS) defined accordingly to GINA guidelines, long acting muscarinic antagonists (LAMA) and oral corticosteroids (OCS) which dose were expressed as daily dose of prednisone equivalent.

Statistical analysis

Continuous variables were expressed as mean ± standard deviation (SD), while categorical variables were reported as counts and percentages. To assess differences in variables measured repeatedly over time, the Wilcoxon signed-rank test was used for continuous data, and the Chi-square or Fisher's exact test (for groups with $n < 5$) was applied for categorical data. Comparisons between independent groups were performed using the t-test for normally distributed continuous variables, the Mann–Whitney U test for non-normally distributed continuous variables, and the Chi-square or Fisher's exact test for

categorical variables, as appropriate. Normality of distribution was assessed using the Kolmogorov–Smirnov (KS) test. A multivariate logistic regression analysis was conducted to identify independent predictors of clinical remission at visit 2 (V2); p-values were derived from the Wald test. Receiver Operating Characteristic (ROC) curve analysis was used to evaluate the diagnostic performance of continuous variables in predicting asthma remission at follow-up. The area under the ROC curve (AUC) was calculated as a summary measure of test accuracy. The correlation coefficient and corresponding p-value were calculated using a point-biserial correlation between the continuous variable and the binary outcome. All statistical analyses were performed using IBM SPSS Statistics, version 23.0 (IBM Corp., Armonk, NY, USA). A p-value < 0.05 was considered statistically significant.

Table S1. Comparisons of baseline clinical characteristics between biologic-naïve and switched patients

	**BIOLOGIC NAIVE**	**BIOLOGIC SWITCHED**	**p-value**
Subjects (n)	31	21	-
Age (years)	56.2 ± 18.2	50.4 ± 14.9	n.s.
Males, n (%)	18 (58)	9 (42.8)	n.s.
Former smokers, n (%)	15 (48.3)	7 (33.3)	n.s.
Asthma onset under 18 y, n (%)	13 (41.9)	9 (42.8)	n.s.
Asthma onset over 40 y, n (%)	11 (35.4)	5 (23.8)	n.s.
Atopy, n (%)	21 (67.4)	16 (76.1)	n.s.
PAS, n (%)	16 (51.6)	13 (61.9)	n.s.
SAS, n (%)	5 (16.1)	3 (14.2)	n.s.
Allergic rhinitis, n (%)	20 (64.3)	16 (76.1)	n.s.
Atopic dermatitis, n (%)	8 (25.8)	5 (23.8)	n.s.
CRSwNP, n (%)	18 (58)	12 (57.1)	n.s.
GERD, n (%)	16 (51.6)	9 (42.8)	n.s.
Obesity, n (%)	7 (22.5)	4 (19)	n.s.
ACOS, n (%)	3 (9.6)	0 (0)	n.s.

OSAS, n (%)	5 (16.1)	2 (9.5)	n.s.
Emphysema, n (%)	3 (9.6)	0 (0)	n.s.
Bronchiectasies, n (%)	2 (6.4)	5 (23.8)	n.s.
Hypertension, n (%)	8 (25.8)	1 (4.7)	n.s.
Type 2 diabetes mellitus, n (%)	2 (6.4)	2 (9.5)	n.s.
ACT (pts)	16 ± 6	18 ± 6	n.s.
AE rate (ep/months)	0.17 ± 0.20	0.12 ± 0.23	n.s.
Serious AE (n)	0.48 ± 0.76	0.14 ± 0.35	n.s.
FVC (lt)	3.577 ± 1.156	3.661 ± 1.148	n.s.
FEV1 (lt)	2.345 ± .959	2.385 ± .922	n.s.
FEF_{2575} (lt)	1.377 ± 1.059	1.506 ± .855	n.s.
FEV1/FVC	64.3 ± 13.4	64.4 ± 9.8	n.s.
Blood eosinophils (cell/µl)	439 ± 314	474 ± 364	n.s.
Total serum IgE (kU/L)	723 ± 1439	1438 ± 1882	n.s.
FeNO > 25 ppb, n (%)	8 (53.3)*	5 (83)*	n.s.
High dose ICS, n (%)	27 (87)	17 (80)	n.s.
Manteniance OCS, n (%)	14 (45.1)	10 (47.6)	n.s.
Daily prednisone equivalence (mg)	5.6 ± 8.1	4.6 ± 7.2	n.s.
LAMA, n (%)	16 (51.6)	11 (52.6)	n.s.

*Data are reported as mean ± standard deviation for continuous variables or absolute (relative) frequency for nominal variables. The comparison between baseline variables according to remission status at 12 months has been performed with t-test for continuous variables or chi-square test / fisher exact test for nominal variables. n.s. = not significant. 2 SAS=exclusively seasonal allergens sensitization; PAS=perennial allergen sensitization; other abbreviations are reported in the methods and material section. * data available for 22 out of 52 patients*
